# Within-country heterogeneity in patterns of social contact relevant for tuberculosis infection transmission, prevention, and care

**DOI:** 10.1371/journal.pgph.0004257

**Published:** 2025-07-15

**Authors:** Kate E. LeGrand, Anita Edwards, Mbali Mohlamonyane, Njabulo Dayi, Stephen Olivier, Dickman Gareta, Robin Wood, Alison D. Grant, Richard G. White, Keren Middelkoop, Palwasha Khan, Nicky McCreesh

**Affiliations:** 1 TB Centre, London School of Hygiene & Tropical Medicine, London, United Kingdom of Great Britain and Northern Ireland; 2 Africa Health Research Institute, KwaZulu-Natal, South Africa; 3 Desmond Tutu HIV Centre, Department of Medicine, University of Cape Town, Cape Town, South Africa; 4 Institute of Infectious Disease and Molecular Medicine, University of Cape Town, Cape Town, South Africa; 5 School of Laboratory Medicine and Medical Sciences, University of KwaZulu-Natal, Durban, South Africa; Boston University, UNITED STATES OF AMERICA

## Abstract

*Mycobacterium tuberculosis* (*Mtb*) transmission is driven by variable social, environmental, and biological factors, including the number and duration of indoor contacts. Social contact data can provide information on potential transmission patterns, but is underutilised outside the field of mathematical modelling. We explore three contexts where contact data can provide valuable insights: 1) household contact tracing; 2) infection prevention and control measures (IPC); and 3) contamination in cluster randomised trials (CRTs). A social contact survey was conducted in adults aged 18 and older from three communities with comparable population sizes in South Africa: an urban township and peri-urban and rural clinic catchment areas. Participants reported congregate settings visited over 24-hours, visit durations, and estimated number of people present. To correspond with the three contexts, we estimated the proportion of contact hours occurring 1) within the home; 2) in congregate settings outside the home; and 3) outside the participants’ communities. Participants reported a mean of 27.0 (rural), 55.2 (peri-urban), and 73.0 (urban) contact hours. The proportions of household contact were similar among rural and peri-urban participants (76.8% and 71.7%), compared to urban (48.6%). Congregate settings visited varied; urban participants spent the most contact hours in retail/office settings (19.9%), peri-urban participants in community-service buildings (20.4%), and rural participants in other peoples’ homes (25.5%). Urban participants reported the highest proportion of contact outside the community (67.0%) compared to rural (38.8%) and peri-urban (21.5%) participants. The observed heterogeneity in contact patterns has implications for TB interventions. Household contact tracing may be most effective in the rural community where household contact was highest. The diverse range of congregate settings visited suggests that prioritising IPC measures in these locations may enhance their overall efficacy. Considering contact patterns when designing clusters may reduce contamination in CRTs. Tailored interventions, informed by local contexts, are essential to reduce TB burden.

## Introduction

Tuberculosis (TB) is a pervasive threat to global public health. In 2023, an estimated 10.8 million developed TB, and an estimated 1.25 million people died from the disease [1]. South Africa contends with a particularly high TB burden with a person developing incident TB every two minutes, while every nine minutes the disease claims another life [1,2]. Reducing TB burden requires disrupting the transmission of *Mycobacterium tuberculosis* (*Mtb*), the bacillus that causes the majority of TB disease.

*Mtb* spreads from person to person through airborne transmission, influenced by highly variable social, environmental, and biological factors, including the frequency and duration of social contact, as well as the time spent in poorly ventilated spaces [3]. While sustained household exposure has historically been considered the primary route for transmission, recent molecular and epidemiological evidence suggests that more than 80% of *Mtb* transmission in high burden settings is attributable to exposure outside the household [4–7]. Identifying congregate settings where transmission may occur requires a comprehensive understanding of contact patterns within the broader population. Such investigations are pivotal for elucidating the dynamics of *Mtb* transmission and developing effective strategies.

The incubation period from the acquisition of *Mtb* infection to the onset of TB disease poses a challenge for intervention implementation. Approximately 5% of healthy adults who acquire *Mtb* infection will progress to TB disease within the first two years, while others may develop the disease a decade or longer after exposure [8,9]. This latency makes it difficult to identify the propitious conditions for transmission. Social contact surveys can partly address this limitation by capturing detailed information on social behaviours and population movement. These surveys typically inquire about close contacts (face-to-face conversations or physical touch) [10,11]. While less common, some surveys also include data on casual contacts (people ‘sharing air’ in indoor settings), which may be more relevant for airborne infections like *Mtb* [12,13]. While social contact data offer valuable insights, contact patterns can differ systematically across settings, which has implications for understanding the dynamics of transmission [10,14,15].

Given the heterogeneity of contact patterns across populations, a uniform approach for interventions may be insufficient to interrupt transmission. To bridge the knowledge gap between social contact patterns and their implications for TB interventions, we analysed data from a social contact survey conducted among adults in three geographically distinct communities in South Africa. We examined three TB contexts where social contact data can provide valuable insights: Context 1) Household contact tracing, where we estimate the proportion of contact hours occurring within participants’ own homes; Context 2) Infection prevention and control strategies, where we estimate the proportion of contact hours occurring in various congregate settings; and Context 3) Contamination in cluster randomised trials, where we estimate the proportion of contact hours occurring outside the community. For this third context, we considered the communities as clusters in a trial, and contact outside the community as a measure of mobility. Contamination in CRTs, resulting from mobility between clusters and the wider population, can dilute the intervention impact and lead to an underestimation of the true effect [16].

## Materials and methods

### Ethics statement

This study is a secondary analysis of social contact survey data collected from human participants. The survey protocol was approved by ethics committees at the London School of Hygiene and Tropical Medicine (LSHTM) (14520 & 14640), University of KwaZulu-Natal (UKZN) (BE662/17), and University of Cape Town (008/2018). Ethics approval for this secondary analysis was granted by LSHTM (28263) and UKZN (BREC/00005202/2023).

### Study communities

**Fig 1:**
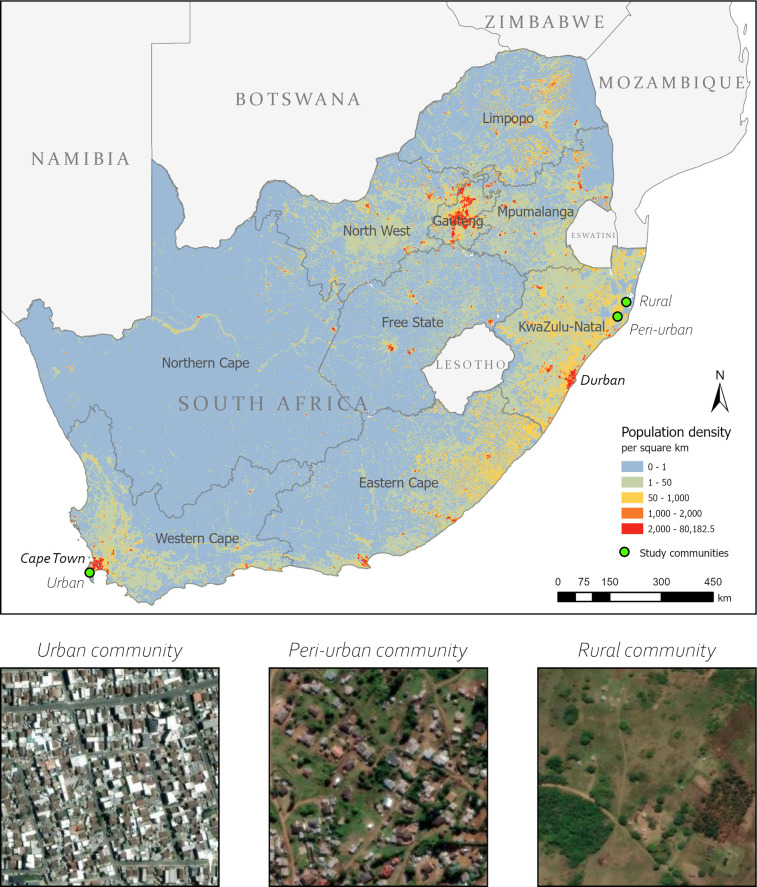
Map of study community locations and population densities in South Africa, 2020. km = kilometre. Area of each satellite imagery inset is approximately 0.1 km^2^. Administrative boundary shapefiles from geoBoundaries [24], satellite imagery from ESRI [25], and national population density from WorldPop [26].

In 2019, a social contact survey was conducted in three communities in South Africa with comparable population sizes: an urban township in Western Cape (WC) province and peri-urban and rural areas in KwaZulu-Natal (KZN) province (Fig 1) [17]. These communities were selected because of their established partnerships with local academic and research institutions and demographic surveillance, which facilitated data collection. While high TB notification rates and HIV prevalence dominate the public health landscape of the three study communities, these strong collaborations facilitate research and programmes for care and prevention [18–20].

The urban community is an established research site with biennial censuses, located approximately 40 kilometres (km) south of Cape Town. This well-demarcated township has an area of about 0.5 km^2^ and a population of 27,000, making it the most densely populated community in this study (54,000 people/km^2^) [17]. The community comprises a formal sector with numbered housing plots with basic utilities, and an informal sector with crowded shack dwellings and limited communal services, which are characteristic of low socioeconomic communities [21]. The weighted mean household size in the urban community varied by definition, with 3.9 people per dwelling among survey participants and 13.2 people per plot among community members in the census.

The peri-urban and rural communities are each a catchment area of a primary healthcare clinic within a health and demographic surveillance area. In the overall surveillance area, most households have access to basic utilities with over 95% of households having electricity and toilets, and 66% have access to piped water [22]. At their closest point, the peri-urban and rural communities are approximately 12 kilometres apart. The peri-urban community spans an area of about 165 km^2^ with a population of 25,000 (population density of 152 people/km^2^). The rural community has the lowest population density of the three communities (85 people/km^2^) with a population of approximately 22,000 and an area of 260 km^2^. Both communities have large household sizes, with peri-urban and rural participants reporting a weighted mean of 15.2 and 10.9 people per plot, respectively. These households reflect all people living on the same plot, which may contain multiple dwellings [23], as data on individual dwellings were not available.

### Survey design and data collection

In the urban community (WC), data were collected between 14 May 2019 and 15 October 2019. 1,530 adults aged 15 and older were selected from an estimated population of 27,000 by using age- and sex-stratified random sampling, based on a census conducted among residents in the demographic surveillance area shortly before the study. Up to five contact attempts were made to recruit participants.

In the peri-urban and rural communities (KZN), data were collected from 28 March 2019 to 9 December 2019. A random sampling approach was used to select 3,093 adults aged 18 and older registered as residents in the demographic surveillance area. Sampling was stratified by residential area (each containing about 350 households), with selection probability weighted by the number of eligible residents per area, based on a census conducted area by area in advance of the social contact survey. Each potential participant in KZN received up to three contact attempts.

For this comparative analysis, we excluded respondents from WC aged 15 to 17, as the KZN survey collected data from people aged 18 and older only. The methods for survey design and data collection have been previously described in full [17].

Participants were interviewed in-person at their homes and asked to recall their activities on a randomly assigned day in the past week in the KZN communities, and on the day preceding the interview in the WC community. Participants listed all buildings visited, and for each building the type of building, the location of the building, the duration of the visit, and the estimated number of people present halfway through the visit. Additionally, participants reported information about their use of transportation, including the vehicle type, duration, and number of people present at the start of their journey. Sociodemographic data, including age, sex, and employment status, were also collected. Details on the survey questions analysed for this study are available in the Supporting information (S1 Appendix).

All data used in this secondary analysis were received by 29 May 2024 and were fully anonymised before access. Informed consent, including permission to share anonymised data for future studies, was obtained from all participants by the original survey investigators.

### Statistical analysis

We analysed the social contact survey data to assess the potential implications of contact patterns on the effectiveness and evaluation of tuberculosis interventions. We classified the location of contact as relevant for three TB contexts: Context 1) Household contact tracing, where we estimate the proportion of contact time occurring within participants’ homes; Context 2) Infection prevention and control settings, where we estimate the proportion of contact time occurring in different indoor congregate settings; and Context 3) Contamination in cluster randomised trials, where we estimate the proportion of contact time occurring outside the community as a measure of mobility. The WC survey asked whether the building visited was located within the community, whereas the KZN survey asked participants to name the administrative unit within which the building was located. The locations of the KZN buildings were validated using ESRI ArcMap (Version 10.8.1) (see S1 Appendix for further details). Data cleaning, analysis, and visualisation of results were carried out in R.

For airborne infections like *Mtb* where transmission can occur in shared air without close contact, reporting the number of close contacts (i.e. people spoken to or touched) alone does not capture the total amount of exposure an individual has to others. To address this limitation, we estimated cumulative indoor contact hours as the product of the duration of each building visit or transport journey and the number of people present. We set a maximum of 20 people in any building type, and 10 for private cars, as it is unlikely that a participant could have sufficient contact with every adult above this value to allow infection transmission. To account for the potential effects of larger group sizes, we conducted sensitivity analyses with caps of 50 and 100 people in buildings (Table S3 in S1 Appendix). Contact hours were weighted for each community according to the study population composition by age group (18–24, 25–44, 45+) and sex. To account for the differences in the proportions of participants who recalled their activities on each day of the week or weekends, contact hours were also weighted by day of the week. Missing values for the number of people present (urban: 15.3%, n = 148; peri-urban: 2.1%, n = 18; rural: 0.5%, n = 4) and duration of visit (urban: 0.2%, n = 2; peri-urban: 0.1%, n = 1) were imputed by averaging the value from the same conditions (i.e., same community and type of building visited or transport used).

We estimated the number of indoor contact hours for each location setting by community and sociodemographic strata (sex, age, group, and employment status). To understand average contact trends, we calculated the mean number of indoor contact hours (MICH) by taking the sum of contact hours reported for each stratum and dividing it by the total number of participants from that stratum. We then calculated the proportion of contact hours that occurred within the home, outside the home by congregate setting, and outside the community. We generated 95% plausible ranges of MICH and proportions by bootstrapping. Missing values for the number of people present and the duration of the visit were imputed by selecting a random value given the same conditions for each bootstrap iteration (S1 Appendix).

While absolute mean contact hours will provide some indication of the risk of transmission by location in each community, differences in TB prevalence between communities will influence transmission risk, making direct comparisons between communities challenging. We therefore report proportions of contact hours to facilitate comparisons. For example, a higher proportion of contact hours in municipal buildings would suggest that targeted IPC measures in these buildings could result in a greater relative reduction in TB incidence in that community. By comparing the proportions of contact hours across communities, we can identify interventions that may be most contextually relevant and impactful.

The impact of household contact tracing on *Mtb* transmission in the wider population may be limited, and may vary between settings [27]. In Context 1, to assess the potential for household contact tracing, we compared the proportion of contact hours occurring within and outside a participant’s own home.

Contact patterns outside the household are particularly relevant in understanding how TB spreads, and are useful for informing the implementation of IPC interventions in congregate settings [28]. In Context 2, we examined the proportion of contact hours spent outside the home in ten groupings of buildings and transport: community services (e.g., clinics, hospitals, churches, libraries), food and leisure, retail and office, schools, workshops (e.g., mechanic shops, factories), own homes, other homes, other buildings, unknown buildings, and an overall transport category. A full list of these groupings is available in the Supporting information (Table S1 in S1 Appendix).

Understanding the geographic location of where contact occurs can have an effect on the design and evaluation of CRTs [29]. Contamination due to mobility between clusters and the wider population can dilute the impact of interventions, leading to an underestimation of their true effect [16]. In Context 3, we considered the communities in this study as clusters and analysed the proportion of contact hours spent outside each community as a measure of mobility, which can be an indicator of contamination. As it was not possible to definitively determine whether contacts made during transport were with individuals from within or outside the community, contact hours in transport were excluded from the proportion calculations in Context 3.

## Results

### Sociodemographic characteristics of survey participants

A summary of the included participants is provided in Table 1. A total of 4,623 individuals were sampled across the three study communities resulting in 2,673 (58%) participants in the analysis.

**Table 1:** Sociodemographic characteristics of survey participants.

Participant characteristics	Urban		Peri-urban		Rural	
	Count	Percentage	Count	Percentage	Count	Percentage
**Sex**						
Female	481	49.6	466	55.3	485	56.3
Male	489	50.4	376	44.7	376	43.7
**Age group**						
18-19	76	7.84	63	7.48	55	6.39
20-29	376	38.8	244	29.0	251	29.2
30-39	333	34.3	152	18.1	155	18.0
40-49	133	13.7	123	14.6	104	12.1
50+	52	5.36	260	30.9	296	34.4
**Mean household size**						
Individuals in own dwelling	3.9	NA	NA	NA	NA	NA
Individuals on same plot	13.2	NA	15.2	NA	10.9	NA
**Time lived in house**						
Less than 2 years	224	23.1	20	2.38	45	5.23
2-5 years	234	24.1	76	9.03	21	2.44
More than 5 years	511	52.7	746	88.6	795	92.3
Unknown	1	0.103	0	0	0	0
**Time lived in community**						
Less than 2 years	85	8.76	9	1.07	4	0.465
2-5 years	166	17.1	38	4.51	19	2.21
More than 5 years	718	74.0	795	94.4	838	97.3
Unknown	1	0.103	0	0	0	0
**Employment status**						
Full-time	379	39.1	237	28.1	91	10.6
Part-time or casual	191	19.7	33	3.92	35	4.07
Not employed	393	40.5	452	53.7	644	74.8
Unknown	7	0.722	120	14.3	91	10.6
**Day reported**						
Monday	173	17.8	106	12.6	133	15.4
Tuesday	180	18.6	130	15.4	112	13.0
Wednesday	166	17.1	117	13.9	122	14.2
Thursday	119	12.3	139	16.5	111	12.9
Friday	70	7.22	124	14.7	137	15.9
Saturday	83	8.56	119	14.1	126	14.6
Sunday	179	18.5	107	12.7	120	13.9
**Total participants**	970		842		861	

Mean household size is participant-weighted; Household size for a participant’s own dwelling was only available for the urban community; Urban household size per plot is for all households in the community, not for the social contact participants alone; NA = Not available.

Of the 1,530 individuals sampled in Western Cape, 1,213 (79%) were recruited. The remainder had incorrect census information (13%, n = 193), moved or died (8%, n = 117), refused participation (5%, n = 77), or were uncontactable after five attempts (<1%, n = 4). Among those contacted, 14 (1%) were excluded because they were ineligible or because they spoke another language. Technical issues resulted in data loss from 8 interviews, leaving 1,115 (73%) complete interviews. After excluding participants under age 18 or those who were missing age data (10%, n = 145), the final analysis included 970 adults (87% of complete interviews).

Of the 3,093 individuals sampled in KwaZulu-Natal, 1,704 (55%) completed an interview, though one interview was excluded due to data quality concerns. The remaining sampled individuals were either unreachable after three attempts (35%, n = 1,071) or had died or relocated (10%, n = 299). Of the 1,703 included interviews, 842 (49%) and 861 (51%) were from the peri-urban and rural communities, respectively.

The survey participants were mostly representative of each community’s age and sex distribution, except for higher participation among urban 18 to 19 year-olds and lower participation among peri-urban 30 to 39 year-olds (Table S2 in S1 Appendix). In the urban community, participants were younger with 80.9% (785/970) under age 40, compared to 54.5% (459/842) in the peri-urban and 53.5% (461/861) in the rural communities, reflecting the demographics of the study populations (Table S2 in S1 Appendix).

High unemployment was reported by all three communities; urban participants reported the lowest proportion at 40.5% (393/970), compared to nearly three quarters of rural participants (74.8%, 644/861), and more than half of peri-urban participants (53.7%, 452/842). In KZN, the proportions of participants who were employed full-time (19.3%) and part-time (4.1%) were similar to the proportions in the sampling frame (19.5% and 3.8%, respectively). Comparable employment data were unavailable for the WC population.

Nearly all peri-urban (94.4%, 795/842) and rural (97.3%, 838/861) participants were long-term residents of their communities (five or more years), compared to 74.0% (718/970) of urban participants.

### Mean indoor contact hours

**Fig 2:**
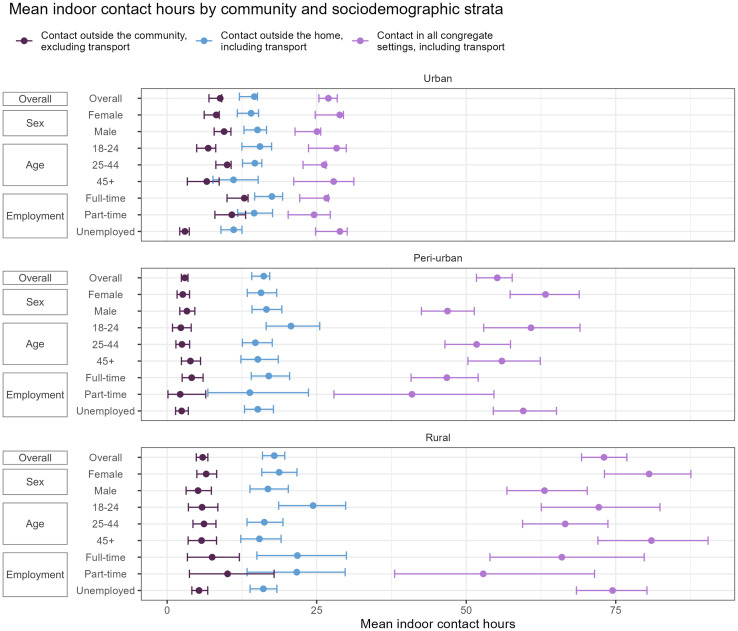
Mean indoor contact hours reported by community and sociodemographic strata. Contact in all congregate settings also includes participants' own homes.

Our analysis of social contact data across the three communities revealed distinct contact patterns overall and by sociodemographic strata (Fig 2; Table S3 in S1 Appendix). Considering all congregate settings, participants from the rural community reported the highest MICH of 73.0 hours (95% CI: 69.3-76.9). This was followed by peri-urban participants, who reported a MICH of 55.2 (CI: 51.7-57.7). Urban participants reported the lowest MICH, 27.0 (CI: 25.4-28.4). Rural participants generally reported the highest MICH across the strata, with the highest among ages 45 and older (81.0, CI: 72.0-90.4). Participants from the urban community reported the lowest MICH across all strata, with part-time employees showing the lowest (24.6, CI: 20.2-27.3)

Contact patterns in congregate settings outside the home were more consistent across the communities. Rural participants had the highest MICH, 17.9 (CI: 15.9-19.7). Peri-urban was similar, with participants reporting a mean of 16.1 (CI: 14.1-17.2), and urban participants had the lowest MICH (14.6, CI: 12.1-15.1). These trends extended to the strata, where rural participants generally reported higher means than the other two communities. Rural participants ages 18–24 reported the highest MICH (24.4, CI: 18.7-29.8). Additionally, full-time rural employees reported the next highest MICH (21.8, CI: 15.0-30.0), surpassing those from the urban (17.5, CI: 14.6-19.3) and peri-urban (17.0, CI: 14.1-20.5) communities.

Disparities in contact patterns emerged when examining contact occurring exclusively outside the community. Urban participants reported the highest MICH (8.9, CI: 7.0-9.1), followed by rural participants (5.9, CI: 4.9-6.8), and peri-urban participants (3.0, CI: 2.4-3.5). Regarding the strata, full-time (12.9, CI: 10.0-13.5) and part-time (10.8; CI: 8.0-13.1) employees from the urban community reported the highest MICH. There were slight differences in MICH by sex, with males having higher means than females in urban and peri-urban settings. Urban males had a MICH of 9.5 (CI: 7.9-10.7) compared to females with 8.2 (CI: 6.2-8.7). In the peri-urban community, these values were 3.3 (CI: 2.1-4.6) and 2.6 (CI: 1.7-3.7), respectively. Rural females reported a MICH of 6.5 (CI: 5.0-8.3) compared to males with a mean of 5.2 (CI: 3.2-7.4).

### Context 1: Household contact tracing

**Fig 3:**
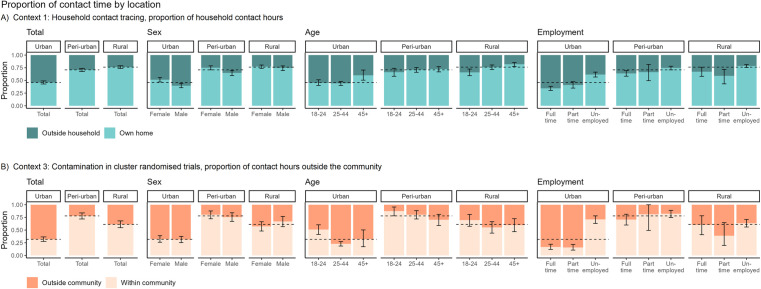
Proportion of contact hours occurring inside and outside households and communities. Dashed lines indicate the overall mean proportion in each community. A) All building types and transport were included for congregate settings outside the household. B) Transport was excluded from contact occurring inside and outside the community.

Participants across the three communities generally spent more contact hours in their own homes than outside the home (Fig 3A). However, there were notable differences in these proportions. Rural participants had the highest proportion of household contact hours (76.2%, CI: 73.5%-79.0%), followed by the peri-urban participants (70.8%, CI: 67.6%-73.8%). By contrast, urban participants spent less than half of contact hours in the household (46.0%, CI: 42.9%-49.0%). Contact hours outside the home were highest in the urban community across all sociodemographic strata, with full-time employees having the greatest proportion (65.8%, CI: 61.7%-69.8%).

### Context 2: Infection prevention and control strategies

**Fig 4:**
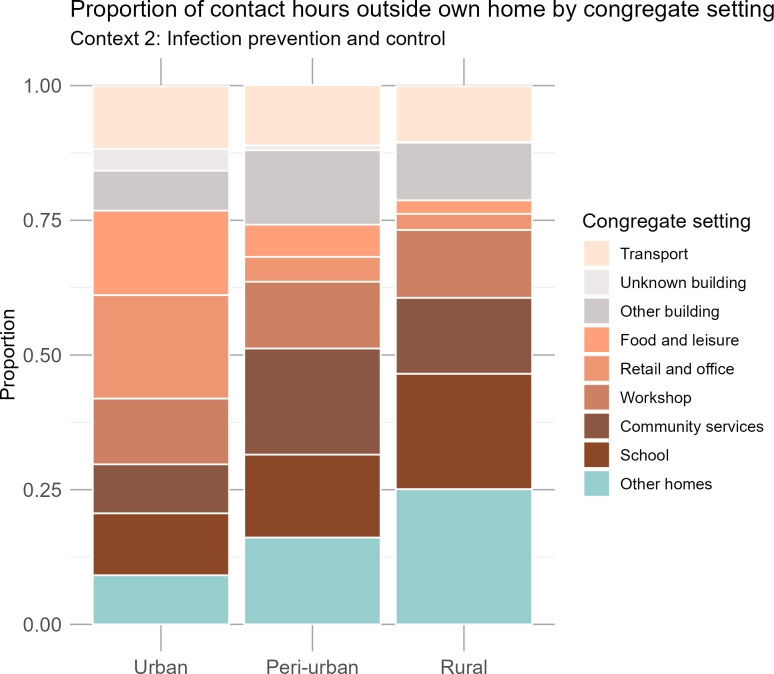
Proportion of indoor contact hours attributable to each congregate setting outside the household.

The proportion of contact hours spent in specific congregate settings outside the home varied substantially across the urban, peri-urban, and rural communities (Fig 4; Table S7 in S1 Appendix). Urban participants had a more diverse range of activities and interactions outside their homes; retail and office buildings accounted for the largest proportion of contact hours (19.2%, CI: 13.8%-21.8%), followed by food and leisure settings (15.7%, CI: 12.8-20.3%). Other people’s homes, community services, and schools each accounted for fewer than 10% of contact hours from urban participants. By contrast, the highest proportion of contact hours among rural participants was within other people’s homes (25.1%, CI: 20.1%-30.5%) and in school (21.4%, CI: 16.3-27.4%). In peri-urban areas the greatest proportion of contact hours occurred in buildings providing community services (19.7%, CI: 15.7%-24.5%), followed by time in other people’s homes (16.1%, CI: 12.3%-20.5%). Contact hours reported in transport were relatively similar across communities, with urban participants reporting the largest proportion (11.7%, CI: 9.9%-15.0%), closely followed by peri-urban (11.2%, CI: 9.1%-13.7%) and rural (10.4%, CI: 8.3%-13.0%) participants.

### Context 3: Contamination in cluster randomised trials

The differences in contact patterns were more pronounced when examining the proportion of contact hours spent within and outside the communities (Fig 3B). Urban participants reported the highest proportion of contact hours outside the community by far, reaching 68.2% (CI: 63.5%-72.4%). This finding stands in stark contrast to participants from the rural (38.8%, CI: 31.9%-45.2%) and peri-urban (22.1%, CI: 16.1%-28.1%) participants who mostly had local interactions. Part-time and full-time employees from the urban community spent an overwhelming proportion of contact hours outside their community, 84.3% (CI: 78.3-89.5%) and 83.4% (77.9-88.1%), respectively. Participants from the peri-urban community spent the least amount of contact time outside the community (17.8%, CI: 11.1-25.2%).

## Discussion

By analysing social contact data from three geographically distinct communities in South Africa, we show a large degree of heterogeneity in contact patterns that could have substantial implications for the design and evaluation of TB interventions.

Our findings suggest that the efficacy of household contact tracing for TB (Context 1) may vary across different geographic settings. Across all three communities, participants reported substantial contact hours at home, consistent with other social contact and epidemiological studies [12,30,31]. While nearly three-quarters of contact hours occurred within homes in peri-urban (70.1%) and rural (76.2%) communities, less than half (46.1%) occurred within urban participants’ homes. While contact saturation may reduce the importance of household contact for overall community-wide transmission [32], household contact studies have shown that the transmission risk to household members increases with longer contact time [33,34]. Therefore, household contact tracing will likely have a greater impact in communities where a higher proportion of contact hours occurs between household members.

When household size was defined as including all individuals on the same plot, mean household sizes were similar across communities: 13.2 people per plot in the urban community, compared to 15.2 and 10.9 in the peri-urban and rural communities, respectively. These larger household sizes often reflect multi-generational family groupings or multi-family households [23]. To account for differences in household definitions (individuals per dwelling and per plot), we analysed contact within participants’ own homes and within the same plot separately, which had a negligible effect on our results (Table S4 in S1 Appendix).

These findings have important implications for household contact tracing. Given the similar household sizes across communities, the observed differences in the proportion of contact hours spent at home likely reflect other factors such as housing density, social behaviours, or economic activities that drive contact outside the home. The higher proportions of household contact in the peri-urban and rural communities suggest that household contact tracing could be a particularly effective strategy for identifying and treating individuals with TB in these communities. Conversely, in the urban community, a substantial portion of contact occurred outside the home, highlighting the greater need for complementary strategies to detect people with TB. When designing and implementing screening programs for TB, it is important to consider the extent of household contact to optimise the effectiveness of these interventions.

Given that high proportions of contact hours occurred in other people’s homes in the peri-urban and rural communities, extending screening beyond household members to include frequent visitors and close contacts could potentially increase yield. A study in Malawi using whole genome sequencing found that household contacts accounted for 8.2% of *Mtb* transmission compared to 1.2% from known non-household contacts [35]. This finding suggests that expanding tracing beyond the home to other known contacts may have limited benefit in high prevalence settings, though effectiveness may vary by epidemiological context. Our results highlight the need for adaptive contact tracing policies that reflect local transmission dynamics.

We evaluated the differences in contact occurring outside the home, as it is relevant for infection prevention and control strategies in congregate settings (Context 2). Our results reinforce findings from other studies that public spaces play an important role in *Mtb* transmission [4–6,14,35,36]. Outside the home, urban participants had the highest proportion of contact hours in retail and office settings (19.2%), while rural participants had the highest proportion of contact hours in other people’s homes (25.1%). The highest proportion of contact hours reported by peri-urban participants occurred in community service buildings such as churches and clinics (19.7%). These findings demonstrate the diversity of contact patterns across different congregate settings, though further research is needed to explore the specific factors that may contribute to *Mtb* transmission in these places.

While contact hours in transport were relatively brief compared to other congregate settings, other studies have shown that overcrowding and poor ventilation in vehicles may increase the risk of *Mtb* transmission [37–39]. In the urban community, Deol et al. found that modes of transport generally had better ventilation compared to buildings [37]. This suggests, that for the urban community, transport may not carry as substantial an infection risk due to ventilation as might be expected. However, further research examining ventilation and transmission risk in transport across diverse community settings is needed to fully understand the role of transport in *Mtb* transmission.

The types of congregate settings visited varied substantially across communities, which underscores the importance of tailoring IPC measures to local contexts. While more expensive IPC methods such as germicidal UV lights and mechanical ventilation can be used in clinical settings [28,40], more accessible approaches like opening windows [37,41], and wearing masks [28,40] could also drastically reduce *Mtb* transmission. Prioritising accessible IPC efforts in high-contact settings may improve their impact on community-wide incidence. For example, encouraging open windows and/or promoting mask use in urban retail and office buildings, peri-urban community buildings, and rural homes could be promising strategies to reduce *Mtb* transmission. By combining social contact surveys with aerobiology and molecular data, future research could help identify the specific congregate settings where transmission is most likely to occur. This information may subsequently be used to target the delivery of IPC interventions where they may have the greatest impact.

Considering each community as a cluster, we analysed contact occurring outside the community to assess mobility, a potential source of contamination in cluster randomised trials (Context 3). Mobility between clusters and the wider population can dilute the intervention impact, leading to a potential underestimation of the true effect [16]. Previous CRTs, including ZAMSTAR and HPTN 071 (PopART), which were conducted in both rural and peri-urban settings, have reported high participant mobility as a limiting factor in demonstrating population-level reductions in TB burden [42,43]. Our social contact analysis reinforces this concern; urban participants spent nearly 70% of their contact hours outside their community, compared to 38.8% and 22.1% among rural and peri-urban participants, respectively. While rural settings may offer some advantages in terms of reduced mobility relative to urban areas, our findings demonstrate that contamination can exist across all geographic contexts and should be systematically assessed.

The observed disparities in contact hours outside the community underscore the importance of considering social contexts in CRTs. The high mobility of urban participants may be specific to this environment, with factors like employment opportunities and proximity to a major city potentially influencing contact and movement patterns. By integrating social contact surveys into trial design, researchers can identify the likely geographic range of mobility, and design clusters based on these patterns to mitigate contamination [29]. Furthermore, mathematical models can incorporate contact survey data to simulate various cluster selection and intervention delivery scenarios, enabling an optimised trial design before significant resources are invested [29].

There are several limitations to our analysis. While this study provides valuable insights into the contact patterns of participants in these three communities, the findings are not intended to be directly generalisable to other populations. Instead, our results highlight that substantial differences in contact patterns can exist between different communities in the same country. Furthermore, the survey methodology and the analyses presented here serve as a framework for assessing contact patterns in other populations. This study also focused exclusively on contact patterns among adults aged 18 and older. Future research incorporating children’s contacts may provide a more comprehensive picture, though the current focus on adult populations captures the primary drivers of community-level *Mtb* transmission.

As with any survey, the social contact data collected were subject to measurement error and potential biases that may have affected the reliability of the data. Participants may have had difficulty remembering and accurately reporting their interactions, including the number of people present, or the time spent in a building or in transport. Estimates of contact hours may also be less accurate when based on a random day in the past week, as was done in KwaZulu-Natal, compared to the day before the interview, as in Western Cape. However, all analyses on contact hours were performed on the dataset weighted by age, sex, and day of the week.

Another limitation was our approach to estimating contact hours. We capped the number of people present in buildings at 20 and in private cars at 10, with no other caps on other modes of transport. Our sensitivity analyses with caps of 50 and 100 people in buildings show a modest effect on contact hours, however the distributions remain similar (Table S3 in S1 Appendix). The survey did not collect data on whether contacts were members of the participant’s household or community, or whether they were residents of a different household or community. We therefore used the locations where contact occurred as a proxy measure (Table S1 in S1 Appendix). Thus, we may have under- or over-estimated the proportion of contact hours that occurred between household members, and with people from outside the community. Additionally, transport was excluded from the contact hour proportions outside the community (Fig 3B). However, given that transport accounted for a minimal share of contact in congregate settings (Fig 4), this exclusion is unlikely to have had a large impact on the results.

The proportion of individuals in KZN who were unreachable after multiple contact attempts (35%), raises important considerations about potential non-response bias. Since the survey was conducted at participants’ homes, those who could not be contacted were likely away, perhaps due to employment, school, social activities, or other travel. This absence may have biased our sample toward people who spent more time at home, resulting in an underestimation of the proportion of contact hours that occur outside the home.

Finally, contact patterns may vary by season. We examined MICH by month to explore potential seasonal trends and observed varying patterns across the three communities (Fig S4 in S1 Appendix). However, the survey was not specifically designed to detect seasonal patterns, which limits our ability to draw conclusions about these observations. Because recruitment was conducted area by area, we cannot differentiate seasonal variation from local differences in contact patterns. Future research should specifically investigate seasonal contact patterns and their implications for *Mtb* transmission.

## Conclusion

Our findings demonstrate the value of social contact data and underscore the importance of tailoring interventions to specific community contexts, as a uniform approach is insufficient to reduce TB. Household contact tracing may be most effective in the rural community where household contact hours were the highest. IPC measures are likely to have the greatest impact on transmission when implemented in congregate settings with high contact hours, and these settings varied by community. In the urban community, retail and office settings were the primary locations for contact outside the home, compared to buildings providing community services and other people’s homes in peri-urban and rural communities, respectively. The potential for contamination in cluster randomised trials may be the greatest in the urban community due to the high mobility reported by urban participants. These findings show the value of social contact surveys and their application for developing locally informed interventions to reduce *Mtb* transmission. By leveraging this knowledge, policymakers and public health practitioners can develop more effective and equitable strategies to end TB in South Africa and other high burden settings.

## Supporting information

S1 Appendix Supplemental methods and results. (PDF)
