# Supplementary material for: Within-country heterogeneity in patterns of social contact relevant for tuberculosis infection transmission, prevention, and care
Source: PLOS Glob Public Health. 2025 Jul 15;5(7):e0004257. doi: 10.1371/journal.pgph.0004257 (PMC12262862; doi:10.1371/journal.pgph.0004257)
Supplement: S1 Appendix — (PDF) [file pgph.0004257.s001.pdf]

Within-country heterogeneity in patterns of social contact relevant for tuberculosis infection transmission, prevention, and care

S1 Appendix. Supplemental methods and results

Kate E. LeGrand et al.

Contents

List of tables ... 2
List of figures ... 2
Methods ... 3
  Ethics ... 3
  Study communities ... 3
  Buildings ... 3
  Transport ... 6
  Sociodemographic strata ... 6
  Contact hours ... 8
  Bootstrapping ... 8
  Sensitivity analyses ... 8
Results ... 9
  Survey representativeness ... 9
  Mean indoor contact hours ... 10
  Sensitivity analyses ... 15
  Proportion of indoor contact hours by location ... 19
  Proportion of indoor contact hours by congregate setting ... 21
Acknowledgments ... 22
References ... 24

List of tables

Table S1 in S1 Appendix. Aggregated building groups based on survey response options. 4

Table S2 in S1 Appendix. Observed survey population percentages compared to the survey sampling frame. 9

Table S3-1 in S1 Appendix. Mean indoor contact hours in the urban community by sociodemographic strata. 12

Table S3-2 in S1 Appendix. Mean indoor contact hours in the peri-urban community by sociodemographic strata. 13

Table S3-3 in S1 Appendix. Mean indoor contact hours in the rural community by sociodemographic strata. 14

Table S4 in S1 Appendix. Comparison of contact patterns by household definition. 15

Table S5-1 in S1 Appendix. Mean indoor contact hours in the urban community with person caps of 20, 50 and 100 in buildings and 10 in private cars, by sociodemographic strata. 16

Table S5-2 in S1 Appendix. Mean indoor contact hours in the peri-urban community with person caps of 20, 50 and 100 in buildings and 10 in private cars, by sociodemographic strata. 16

Table S5-3 in S1 Appendix. Mean indoor contact hours in the rural community with person caps of 20, 50 and 100 in buildings and 10 in private cars, by sociodemographic strata. 17

Table S6 in S1 Appendix. Number and proportion of journeys based on origin and destination among peri-urban and rural participants. 19

Table S7-1 in S1 Appendix. Proportion of contact hours inside and outside the household and community among urban participants 19

Table S7-2 in S1 Appendix. Proportion of contact hours inside and outside the household and community among peri-urban participants. 20

Table S7-3 in S1 Appendix. Proportion of contact hours inside and outside the household and community among rural participants. 20

Table S8 in S1 Appendix. Proportion of contact hours in congregate settings outside own home by community. 21

Table S9 in S1 Appendix. Umoya omuhle project members. 22

List of figures

Fig S1 in S1 Appendix. Example of izigodi to catchment assignments in KwaZulu-Natal 5

Fig S2 in S1 Appendix. Decision tree for analysing HIV status. 7

Fig S3 in S1 Appendix. Mean indoor contact hours reported by community, sociodemographic strata, and risk factor. 11

Fig S4 in S1 Appendix. Seasonal trends in mean indoor contact hours by community. 18

Methods

Ethics

Data collection for the social contact surveys received approval from ethics committees at the London School of Hygiene and Tropical Medicine (LSHTM), the University of Cape Town (UCT), and the University of KwaZulu-Natal (UKZN). The Western Cape survey was approved by the UCT Human Research Ethics Committee (HREC/REF: 008/2018) and the LSHTM Observational/Interventions Research Ethics Committee (14520). The KwaZulu-Natal survey was approved by the UKZN Biomedical Research Ethics Committee (BREC) and the LSHTM Observational/Interventions Research Ethics Committee (14640).

Informed consent was obtained from all participants. The consent form indicated that the data collected may be used by other researchers working on tuberculosis and other infectious diseases. Therefore, subsets of de-identified data were published to an online repository and are available upon reasonable request to the principal investigator of the parent studies.[1,2]

This study is a secondary analysis of the social contact surveys conducted in Western Cape and KwaZulu-Natal. All data used in this study were fully anonymised before access. Ethics approval was granted by ethics committees at LSHTM (28263) and UKZN (BREC/00005202/2023). No new surveys or primary data collection activities were conducted for this secondary analysis.

Study communities

The social contact survey analysed for this study was conducted in three communities with comparable population sizes in South Africa: an urban township in Western Cape (WC) province and peri-urban and rural clinic catchment areas in KwaZulu-Natal (KZN) province. The WC survey was conducted between 14 May 2019 and 15 October 2019. The KZN survey was conducted between 28 March 2019 and 9 December 2019. Full details on the survey design and data collection methods have been previously described in McCreesh *et al*.[3]

Buildings

Respondents were asked to list all indoor congregate settings visited and transport used on a randomly assigned day in the past week in the KZN communities, and on the day preceding the interview in the WC community. For each setting visited participants were asked:

- The type of building visited (including their own home)
- Where the building was located
- How long they spent there
- How many people (adults and children) were there, halfway through the time of their visit

There were slight differences in the response options for the types of buildings visited between the KZN and WC surveys. To address these differences, building types were aggregated into larger congregate settings for analysis (Table S1 in S1 Appendix). If the correct location type could not be identified by the participant or interviewer, “other” was selected and details were provided in free text. “Other” buildings were re-classified into the aggregate categories using local expertise and online searches. Responses indicating a building type that could not reasonably be classified as an indoor setting (e.g., gardening) were excluded from the analysis. A spaza is a small, informal convenience store that sells basic goods and necessities typically out of a window to walk-up customers.

Table S1 in S1 Appendix. Aggregated building groups based on survey response options.

Building group	KwaZulu-Natal option	Western Cape options
**Own home**	Own home	Own home
**Other homes**	Other plot<br>Same plot	Other home off your plot<br>Other home on your plot<br>Other home - plot not specified
**School**	School	School - high school<br>School - higher education<br>School - primary school
**Community services**	Church<br>Clinic/Hospital<br>Creche<br>Library<br>Community hall<br>Civic services<br>Counselling centre	Church<br>Clinic/hospital/medical facility<br>Creche/daycare/after school care<br>Library/community hall/youth centre<br>Internet cafe
**Workshop**	Workshop	Workshop
**Retail and office**	Mall/Stores<br>Spaza<br>Office	Mall<br>Shop – clothes or cosmetics<br>Shop – other<br>Shop – spaza<br>Office
**Food and leisure**	Bar/Sport/Nightclub<br>Salon/Barber<br>Gym<br>Restaurant	Bar/shebeen/nightclub<br>Barbershop/Salon<br>Gym<br>Restaurant<br>Food takeaway
**Other building**	Other<br>Guest house<br>Closed tent	Other<br>Guest house
**Unknown building**	Unknown<br>Not provided	Unknown

In the WC survey, participants were asked whether the building was located within or outside the community. In the KZN survey, however, participants were asked to identify the administrative unit (izigodi) in which each building was located. The izigodis were mapped in ESRI ArcMap (Version 10.8.1) and compared to the boundaries of the peri-urban and rural catchment areas. Because the clinic catchment areas are not official administrative units, satellite imagery was used to identify population centres of the izigodis – if the population was largely within catchment area, the izigodi was considered within the community (Fig S1 in S1 Appendix). The validated building locations were then joined to the KZN survey responses.

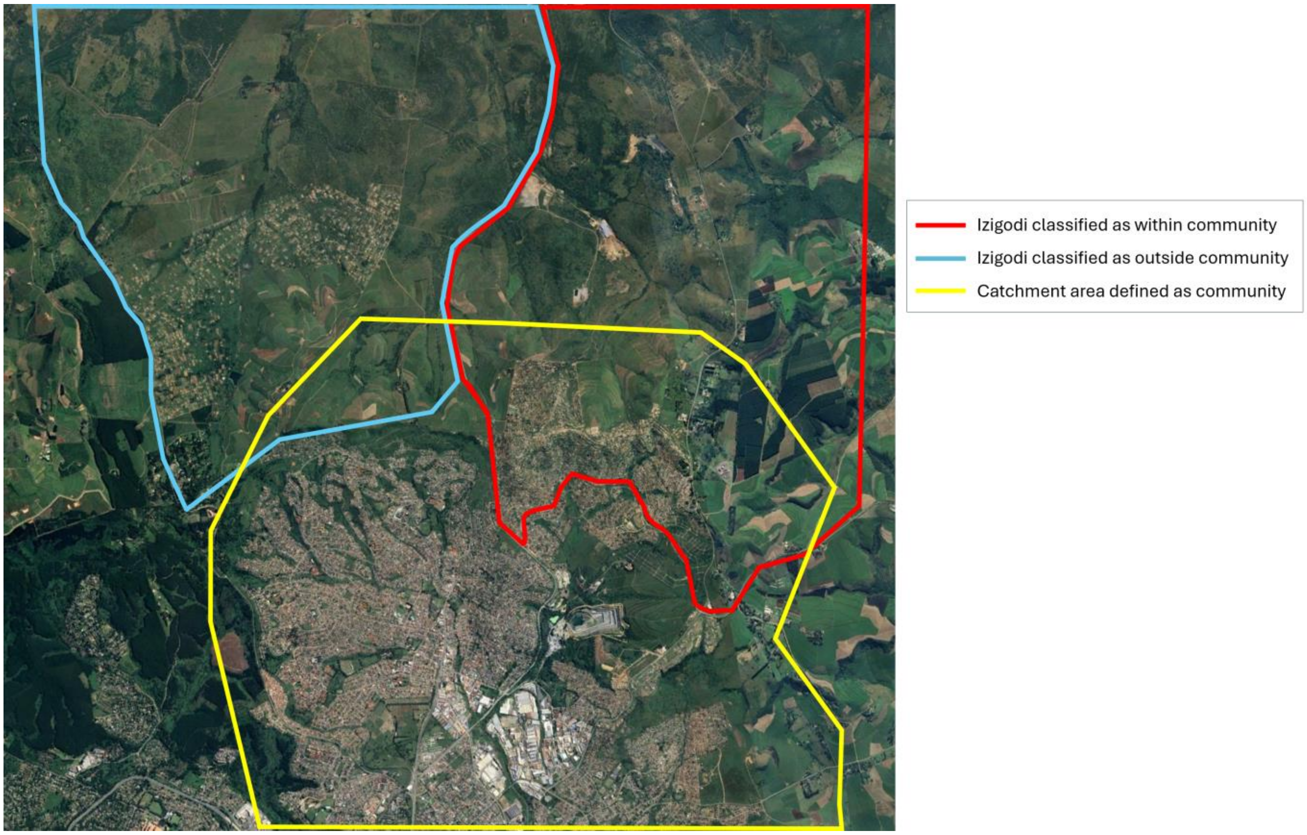

Fig S1 in S1 Appendix. Example of izigodi to catchment assignments in KwaZulu-Natal.

An example clinic catchment area is shown in yellow. The population centre of the red izigodi is located within the boundaries of the catchment area, therefore the izigodi is classified as within the community. While the blue izigodi intersects the catchment area, the population centre is outside, therefore the izigodi is classified as outside the community. This figure is for illustrative purposes only and does not represent boundaries from the KZN study communities.

A limitation of this approach is that contact occurring in buildings within the red izigodi but outside the catchment area would still be classified as within-community contact. However, this is unlikely to have a substantial impact on the results – all participant households were located within their respective catchment areas, and the majority of contact hours occurred at home.

Transport

The survey asked participants about the modes of transportation used with several response options; we grouped all modes into a single “transport” category. In our third intervention context, we analysed the proportion of contact hours spent inside and outside the community as a measure of mobility, which can be a source of potential contamination in a cluster randomised trial. As it was not possible to definitively determine whether contacts made during transport were with individuals from within or outside the community, contact hours in transport were excluded from the “outside community” proportions.

Data on the origin and destination of transport journeys were available for the KZN communities. We calculated the number and proportion of journeys occurring between and within locations inside and outside each community.

Sociodemographic strata

In addition to the primary sociodemographic strata presented in the main results (age, sex, employment status), the social contact survey collected data on HIV status, alcohol consumption, and smoking history.

Smoking history

Participants were asked about their smoking history, and responses were grouped as:

- Currently smoke (Smoker)
  - 10 or more per day
  - 10 or fewer per day
- No smoking or previous history (Non-smoker)
  - Never or I don’t smoke
  - In the past
- Unknown smoking
  - Refuse to answer
  - No response (NA)

Alcohol consumption

Participants were asked about the frequency at which they drink alcohol. Responses were grouped as:

- Any alcohol consumption
  - 4 or more times a week

    - 2-3 times a week
    - 4 or more times a week
    - 2-3 times a month
    - Monthly or less
- No alcohol
    - Never or I don't drink alcohol
- Unknown alcohol
    - Refuse to answer
    - No response (NA)

HIV status

Participants were asked a series of questions about their HIV status from which we categorised responses into positive, negative, and unknown categories (Fig S2 in S1 Appendix). This information was based solely on self-reporting; no HIV testing was conducted as part of the social contact survey. Negative HIV test results were considered valid only if they had been conducted within the past two years. Given the high HIV prevalence in each of the three communities, tests older than two years were classified as unknown.

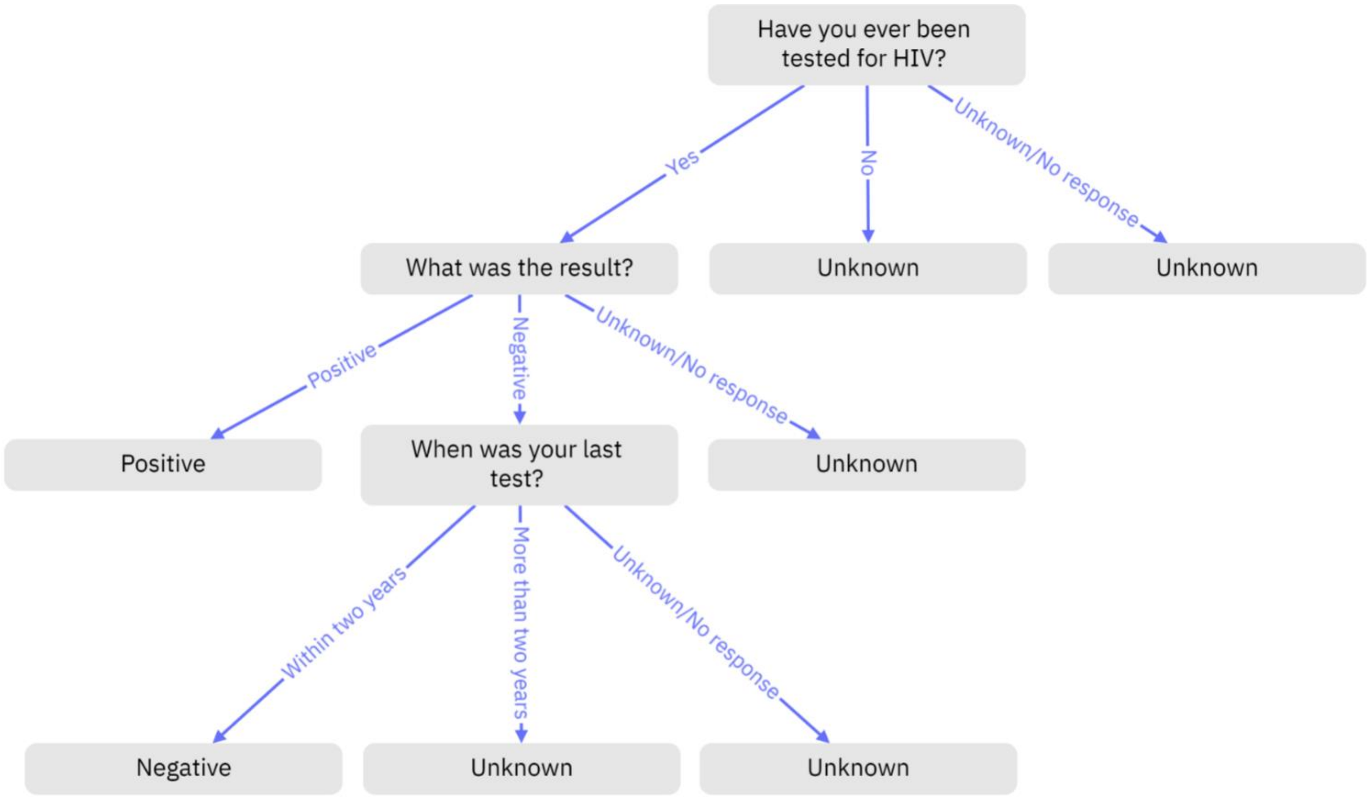

Fig S2 in S1 Appendix. Decision tree for analysing HIV status.

Contact hours

We estimated cumulative contact hours as the product of the duration of each building visit or transport journey and the number of people present. If the duration of time spent in the location was missing, the duration was imputed by averaging the duration from the same conditions (same community and congregate setting type). Missing values in the number of people present were similarly imputed by averaging the value from the same conditions. By using averages, we assume that the missing values are likely to be similar as the observed values in similar scenarios, providing a reasonable estimate given the available information. This approach maintains consistency and representativeness in the data for each community.

Bootstrapping

95% plausible ranges for mean indoor contact hours (MICH) and the contact proportions were generated using 1,000 bootstrapped samples. Respondents were re-sampled with replacement given the same community, sociodemographic strata, and intervention context. During bootstrapping, missing values were replaced with randomly selected values given the same conditions. By using random values, we essentially created multiple hypothetical datasets that could have occurred, allowing for a wider range of possible outcomes in the bootstrapping analysis. This method introduces additional uncertainty, which can better capture the variability in the data and provide more accurate plausible ranges.

Sensitivity analyses

Occupancy

In our main analysis, we implemented a cap of 20 people in buildings and 10 people in private cars. We did not implement a cap on any other type of transportation. These caps were selected for two reasons: first, based on the practical consideration that in high occupancy locations, participants are unlikely to have sufficient and sustained contact with every person above this threshold to allow infection transmission; and second, to address data quality issues where high numbers of people were reported in private cars (n=10), likely reflecting people sitting in the back of pick-up trucks. To explore the uncertainty around reductions in contact with increasing occupancy, we conducted sensitivity analyses with caps of 50 and 100 people in buildings and maintained the cap of 10 in private cars.

Results

Survey representativeness

To evaluate the survey's representativeness, we conducted Z-tests for proportions by comparing the proportion of survey participants and the sampling frame by age and sex groups. Overall, the participants were mostly representative of the population demographics (Table S2 in S1 Appendix). However, two specific groups showed differences between the observed and expected proportions (urban 18 to 19 year-olds and peri-urban 30 to 39 year-olds), meaning the survey sample may not be fully representative of the population.

To assess potential under-recruitment of employed individuals in KZN, we compared employment data between the survey participants and the general population using the census conducted ahead of the contact survey. The results were similar; 19.3% (329/1,703) of participants reported full-time employment compared to 19.5% (5,898/30,259) in the census. There were 4.1% (69/1,703) part-time participants versus to 3.8% (1,139/30,259) in the census. No comparable employment data were available for the WC population.

The survey data were weighted according to each community’s sampling frame (age group and sex), as well as by day of the week, to account for variations in the days on which participants recalled their activities. All analyses of contact hours were performed using this weighted dataset to ensure representation of each community’s population.

Table S2 in S1 Appendix. Observed survey population percentages compared to the survey sampling frame.

	Urban		Peri-urban		Rural	
**Participant characteristics**	Survey results (%)	Sampling frame (%)	Survey results (%)	Sampling frame (%)	Survey results (%)	Sampling frame (%)
**Sex**						
Female	49.6	47.6	55.3	58.4	56.3	62.3
Male	50.4	52.4	44.7	41.6	43.7	37.7
**Age group**						
18-19	7.84	4.69	7.48	5.50	6.39	3.81
20-29	38.8	34.6	29.0	26.8	29.2	31.6
30-39	34.3	38.3	18.1	24.3	18.0	22.3
40-49	13.7	15.6	14.6	16.5	12.1	12.8
50+	5.36	6.83	30.9	26.9	34.4	29.5

Mean indoor contact hours

The MICH for all sociodemographic strata (sex, age, employment status, smoking history, alcohol consumption, and HIV status) by community and location of contact are presented in Fig S3 in S1 Appendix and Tables S3-1:3 in S1 Appendix. The results for overall, sex, age, and employment status (primary strata) correspond to Fig 2 in the main text. Because the additional strata did not show substantial variation in contact patterns compared to the distribution of the primary strata, we opted not to show these additional results in the main findings.

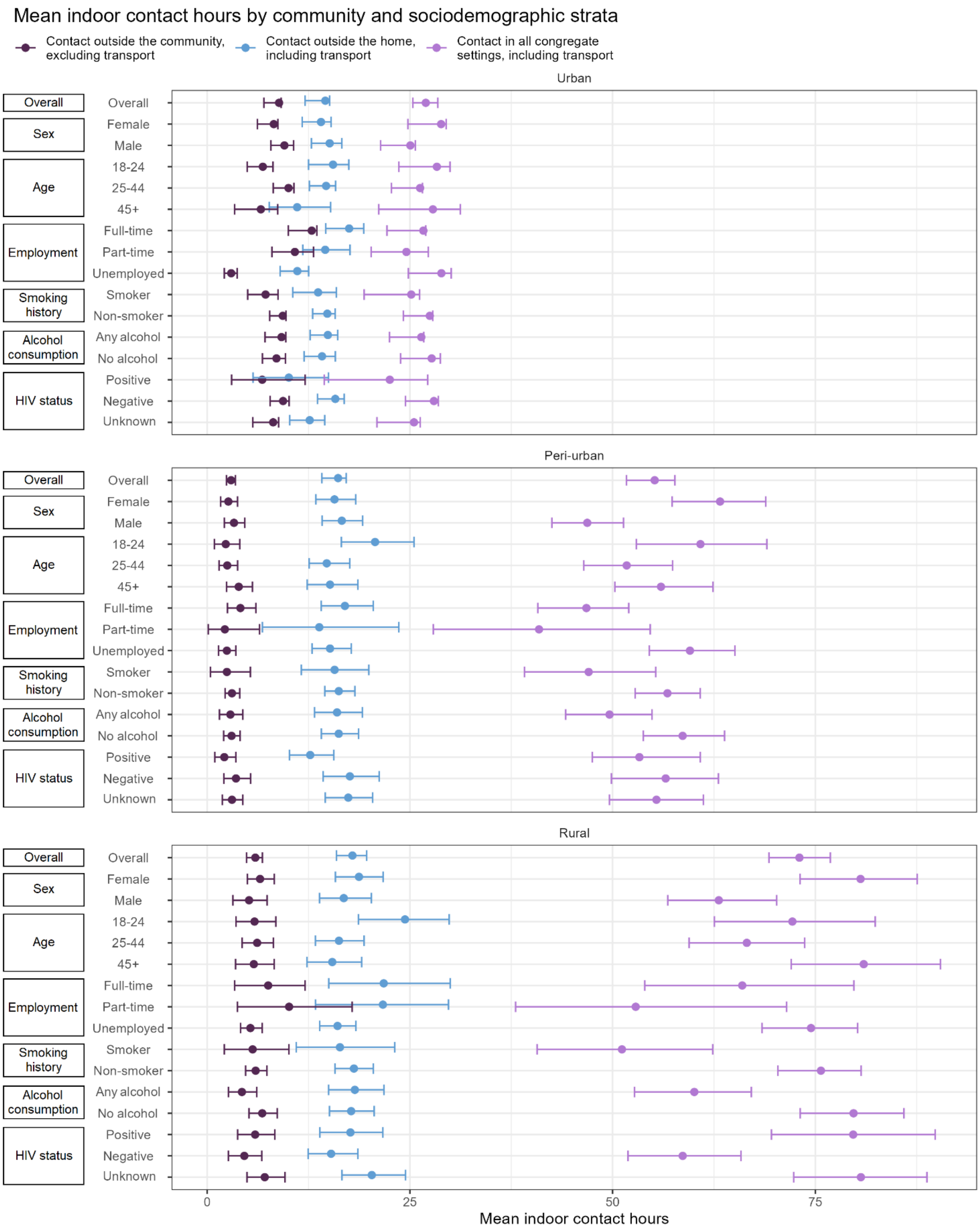

Fig S3 in S1 Appendix. Mean indoor contact hours reported by community, sociodemographic strata, and risk factor.

Table S3-1 in S1 Appendix. Mean indoor contact hours in the urban community by sociodemographic strata.

**Urban community**						
	**Contact in all congregate settings, including transport**		**Contact outside the home, including transport**		**Contact outside the community, excluding transport**	
**Strata**	**MICH**	**95% CI**	**MICH**	**95% CI**	**MICH**	**95% CI**
**Overall**	27.0	(25.4-28.4)	14.6	(12.1-15.1)	8.9	(7.0-9.1)
**Sex**						
Female	28.8	(24.8-29.5)	14.0	(11.7-15.3)	8.2	(6.2-8.7)
Male	25.1	(21.4-25.7)	15.1	(12.9-16.6)	9.5	(7.9-10.7)
**Age group**						
18-24	28.3	(23.6-29.9)	15.5	(12.5-17.5)	6.9	(4.9-8.1)
25-44	26.3	(22.7-26.6)	14.7	(12.6-15.8)	10.0	(8.1-10.7)
45+	27.8	(21.2-31.2)	11.1	(7.7-15.2)	6.6	(3.4-8.7)
**Employment**						
Full-time	26.7	(22.2-27.0)	17.5	(14.6-19.3)	12.9	(10.0-13.5)
Part-time	24.6	(20.2-27.3)	14.6	(11.8-17.6)	10.8	(8.0-13.1)
Unemployed	28.9	(24.8-30.1)	11.1	(9.0-12.5)	3.0	(2.1-3.7)
**Smoking history**						
Smoker	25.2	(19.4-26.2)	13.7	(10.6-15.9)	7.2	(5.0-8.7)
Non-smoker	27.5	(24.2-27.8)	14.8	(13.0-15.8)	9.3	(7.7-9.7)
**Alcohol consumption**						
Any alcohol	26.4	(22.5-26.7)	14.9	(12.7-16.1)	9.2	(7.1-9.7)
No alcohol	27.7	(23.9-28.8)	14.2	(11.9-15.8)	8.5	(6.8-9.7)
**HIV status**						
Positive	22.5	(14.4-27.2)	10.1	(5.7-15.0)	6.8	(3.0-12.1)
Negative	28.0	(24.4-28.5)	15.8	(13.6-16.9)	9.4	(7.8-10.1)
Unknown	25.5	(20.9-26.3)	12.6	(10.2-14.5)	8.1	(5.6-8.8)

Table S3-2 in S1 Appendix. Mean indoor contact hours in the peri-urban community by sociodemographic strata.

**Peri-urban community**						
	**Contact in all congregate settings, including transport**		**Contact outside the home, including transport**		**Contact outside the community, excluding transport**	
**Strata**	**MICH**	**95% CI**	**MICH**	**95% CI**	**MICH**	**95% CI**
**Overall**	55.2	(51.7-57.7)	16.1	(14.1-17.1)	3.0	(2.4-3.5)
**Sex**						
Female	63.3	(57.3-68.9)	15.7	(13.4-18.3)	2.6	(1.7-3.7)
Male	46.9	(42.5-51.3)	16.6	(14.2-19.2)	3.3	(2.1-4.6)
**Age group**						
18-24	60.8	(52.9-69.0)	20.7	(16.5-25.5)	2.3	(0.9-4.0)
25-44	51.7	(46.4-57.4)	14.7	(12.6-17.6)	2.5	(1.5-3.8)
45+	56.0	(50.3-62.4)	15.2	(12.3-18.6)	3.9	(2.4-5.6)
**Employment**						
Full-time	46.8	(40.8-52.0)	17.0	(14.1-20.5)	4.1	(2.5-6.0)
Part-time	40.9	(27.9-54.6)	13.8	(6.8-23.6)	2.2	(0.1-6.5)
Unemployed	59.5	(54.5-65.1)	15.2	(12.9-17.8)	2.4	(1.4-3.5)
**Smoking history**						
Smoker	47.0	(39.1-55.3)	15.7	(11.6-19.9)	2.4	(0.4-5.3)
Non-smoker	56.8	(52.8-60.8)	16.2	(14.5-18.2)	3.1	(2.2-4.0)
**Alcohol consumption**						
Any alcohol	49.6	(44.2-54.9)	16.0	(13.2-19.1)	2.9	(1.5-4.4)
No alcohol	58.6	(53.8-63.8)	16.2	(14.1-18.7)	3.0	(2.0-4.1)
**HIV status**						
Positive	53.3	(47.5-60.8)	12.7	(10.1-15.6)	2.1	(0.9-3.5)
Negative	56.5	(49.8-63.0)	17.6	(14.3-21.2)	3.6	(2.1-5.4)
Unknown	55.4	(49.6-61.2)	17.4	(14.6-20.4)	3.1	(1.9-4.4)

Table S3-3 in S1 Appendix. Mean indoor contact hours in the rural community by sociodemographic strata.

Rural community						
	Contact in all congregate settings, including transport		Contact outside the home, including transport		Contact outside the community, excluding transport	
**Strata**	**MICH**	**95% CI**	**MICH**	**95% CI**	**MICH**	**95% CI**
**Overall**	73.0	(69.3-76.9)	17.9	(15.9-19.7)	5.9	(4.9-6.8)
**Sex**						
Female	80.6	(73.1-87.6)	18.7	(15.8-21.7)	6.5	(5.0-8.3)
Male	63.1	(56.8-70.2)	16.8	(13.9-20.3)	5.2	(3.2-7.4)
**Age group**						
18-24	72.2	(62.6-82.4)	24.4	(18.7-29.8)	5.8	(3.6-8.5)
25-44	66.5	(59.4-73.7)	16.2	(13.4-19.4)	6.2	(4.3-8.2)
45+	81.0	(72.0-90.4)	15.4	(12.3-19.1)	5.8	(3.5-8.3)
**Employment**						
Full-time	66.0	(54.0-79.8)	21.8	(15.0-30.0)	7.5	(3.4-12.1)
Part-time	52.8	(38.0-71.5)	21.7	(13.4-29.8)	10.1	(3.7-17.9)
Unemployed	74.5	(68.4-80.2)	16.1	(13.9-18.3)	5.3	(4.1-6.8)
**Smoking history**						
Smoker	51.2	(40.7-62.3)	16.4	(11.0-23.1)	5.6	(2.1-10.1)
Non-smoker	75.7	(70.4-80.6)	18.1	(15.8-20.5)	6.0	(4.7-7.4)
**Alcohol consumption**						
Any alcohol	60.1	(52.7-67.1)	18.2	(15.0-21.8)	4.3	(2.6-6.1)
No alcohol	79.7	(73.1-85.9)	17.8	(15.1-20.6)	6.8	(5.2-8.6)
**HIV status**						
Positive	79.7	(69.6-89.8)	17.7	(13.9-21.7)	5.9	(3.8-8.3)
Negative	58.6	(51.9-65.8)	15.3	(12.5-18.6)	4.6	(2.6-6.7)
Unknown	80.6	(72.3-88.8)	20.3	(16.6-24.5)	7.1	(4.9-9.6)

Because the contexts were not mutually exclusive (i.e., Context 2 included contact both outside the home and outside the community), we additionally estimated the overall MICH occurring exclusively in the home: Urban (12.4, 95% CI: 11.4-13.3), peri-urban (39.0, CI: 35.9-42.1), rural (55.1, CI: 50.9-59.3), and in any building type exclusively within the community, including own home: Urban (16.5, 95% CI: 15.2-17.5), peri-urban (50.4, CI: 47.1-53.9), rural (65.2, CI: 60.7-69.6).

Sensitivity analyses

Household definition

The availability of data on household sizes varied between the study communities. In WC, we collected data from survey participants on the number of individuals living in one's own dwelling. Additional data were available from the WC census on the number of people living on each plot for the whole community. In KZN, data were only available on the number of people living on the same plot, which may include multiple homes.

The social contact survey asked participants to list the types of buildings they visited (Table S1 in S1 Appendix), which specified own home, house on same plot, house on another plot. In the main analysis, we stratified results by contact occurring in own home and in other homes, regardless of plot location. To account for the differences in household definitions, we compared contact patterns in participants' own homes and within homes on the same plot (Table S4 in S1 Appendix).

Table S4 in S1 Appendix. Comparison of contact patterns by household definition.

Community	Metric	Household definition	
		Own home	Own home + Other home on same plot
Urban	Mean household size	3.9	13.2
	Mean indoor contact hours (MICH)	12.4 (11.4-13.3)	12.5 (11.6-13.5)
	Proportion of contact	45.9%	46.3%
	MICH with each other household member	4.3	3.2
Peri-urban	Mean household size	NA	15.2
	Mean indoor contact hours (MICH)	39.0 (35.9-42.1)	39.3 (36.3-42.3)
	Proportion of contact	70.8%	71.3%
	MICH with each other household member	NA	2.6
Rural	Mean household size	NA	10.9
	Mean indoor contact hours (MICH)	55.1 (50.9-59.3)	55.6 (51.5-59.9)
	Proportion of contact	76.2%	76.9%
	MICH with each other household member	NA	5.1

Mean household size is participant-weighted; Household size for a participant's own dwelling was available only for the urban community; Urban household size on same plot is for all households in the community, not for the social contact participants alone; NA = Not available.

Occupancy

The mean indoor contact analysis presented in the main results considered a cap of 20 people in buildings and 10 people in private cars (Tables S5-1:3 in S1 Appendix). To account for the potential effects of larger groups of people in buildings, we conducted sensitivity analyses with caps of 50 and 100 people in buildings and maintained the cap of 10 in private cars. In Tables S5-1:3 in S1 Appendix, we show the results of this sensitivity analysis for the primary sociodemographic strata for Context 1.

Table S5-1 in S1 Appendix. Mean indoor contact hours in the urban community with person caps of 20, 50 and 100 in buildings and 10 in private cars, by sociodemographic strata.

**Urban community**						
	**Cap of 20**		**Cap of 50**		**Cap of 100**	
**Strata**	**MICH**	**95% CI**	**MICH**	**95% CI**	**MICH**	**95% CI**
**Overall**	27.0	(25.4-28.4)	35.5	(31.2-36.2)	40.8	(35.7-43.0)
**Sex**						
Female	28.8	(24.8-29.5)	37.1	(31.6-38.2)	42.0	(37.5-47.0)
Male	25.1	(21.4-25.7)	33.9	(28.6-36.3)	39.6	(35.3-46.8)
**Age group**						
18-24	28.3	(23.6-29.9)	37.3	(30.2-39.4)	41.4	(36.6-49.8)
25-44	26.3	(22.7-26.6)	35.2	(30.2-36.7)	41.1	(37.1-46.5)
45+	27.8	(21.2-31.2)	32.6	(24.0-37.9)	36.6	(27.1-50.2)
**Employment**						
Full-time	26.7	(22.2-27.0)	35.2	(28.2-36.3)	41.2	(35.3-47.1)
Part-time	24.6	(20.2-27.3)	35.3	(28.3-40.5)	43.1	(34.9-52.6)
Unemployed	28.9	(24.8-30.1)	36.0	(29.9-37.1)	38.6	(35.4-46.3)

Table S5-2 in S1 Appendix. Mean indoor contact hours in the peri-urban community with person caps of 20, 50 and 100 in buildings and 10 in private cars, by sociodemographic strata.

**Peri-urban community**						
	**Cap of 20**		**Cap of 50**		**Cap of 100**	
**Strata**	**MICH**	**95% CI**	**MICH**	**95% CI**	**MICH**	**95% CI**
**Overall**	55.2	(51.7-57.7)	66.7	(62.6-70.7)	75.3	(69.9-81.2)
**Sex**						
Female	63.3	(57.3-68.9)	74.4	(67.1-81.8)	81.2	(72.7-89.9)
Male	46.9	(42.5-51.3)	58.8	(52.5-65.5)	69.3	(60.7-78.3)
**Age group**						
18-24	60.8	(52.9-69.0)	77.8	(66.7-90.1)	86	(72.7-100.5)
25-44	51.7	(46.4-57.4)	60.7	(53.6-67.2)	68.9	(59.9-77.5)
45+	56.0	(50.3-62.4)	67.4	(58.8-76.0)	76.7	(66.5-87.9)
**Employment**						
Full-time	46.8	(40.8-52.0)	57.6	(49.2-65.5)	66.8	(55.8-78.3)
Part-time	40.9	(27.9-54.6)	52.9	(33.1-77.0)	71.2	(37.9-113.4)
Unemployed	59.5	(54.5-65.1)	70.1	(62.9-76.9)	77.3	(69.2-86.2)

Table S5-3 in S1 Appendix. Mean indoor contact hours in the rural community with person caps of 20, 50 and 100 in buildings and 10 in private cars, by sociodemographic strata.

Rural community						
	Cap of 20		Cap of 50		Cap of 100	
**Strata**	**MICH**	**95% CI**	**MICH**	**95% CI**	**MICH**	**95% CI**
**Overall**	73.0	(69.3-76.9)	85.5	(79.9-90.0)	94.5	(87.1-100.1)
**Sex**						
Female	80.6	(73.1-87.6)	94.7	(86.4-103.2)	105.9	(94.6-116.9)
Male	63.1	(56.8-70.2)	73.5	(65.5-82.0)	79.6	(69.6-88.7)
**Age group**						
18-24	72.2	(62.6-82.4)	91.4	(77.5-105.9)	98.2	(83.7-114.9)
25-44	66.5	(59.4-73.7)	76.0	(67.2-84.8)	82.7	(71.6-92.5)
45+	81.0	(72.0-90.4)	92.4	(81.8-104.2)	105.5	(91.0-121.2)
**Employment**						
Full-time	66.0	(54.0-79.8)	80.5	(62.0-99.3)	96.2	(71.0-125.4)
Part-time	52.8	(38.0-71.5)	63.8	(43.4-89.8)	77.1	(42.6-112.8)
Unemployed	74.5	(68.4-80.2)	86.3	(79.6-93.1)	94.5	(85.7-102.5)

Seasonality

Contact patterns may vary by season. We therefore estimated MICH by month to explore temporal trends (Fig S4 in S1 Appendix). Our results show that the rural community had the most pronounced seasonal variation throughout the duration of the study, with MICH ranging from 56.3 hours in April to 119.9 hours in March. However, the March peak should be interpreted cautiously because the rural survey started late in the month (28 March 2019), resulting in only a single observation (n=1) that was included in the analysis. There was less seasonal variation in the peri-urban community, with peaks in August (64.6 hours) and November (76.9 hours), and the lowest MICH in April (45.2 hours). The urban community reported modest seasonal changes with the lowest contact hours in June (20.9 hours) and a steady increase through October (39.2 hours).

Recruitment was conducted area by area in each community. At their closest point, the peri-urban and rural communities were about 12 kilometres apart and likely experienced a similar climate, yet they showed different seasonal patterns. Because the data were collected from each area within the same time period, we cannot reliably distinguish seasonal variations from local differences in KZN or WC. Future research could specifically investigate temporal and seasonal effects of contact patterns on *Mtb* transmission.

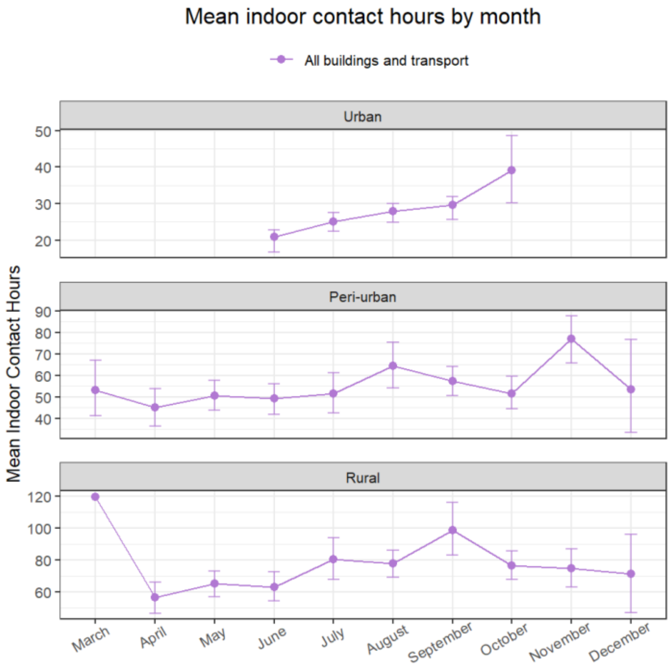

Fig S4 in S1 Appendix. Seasonal trends in mean indoor contact hours by community.
A confidence interval for the rural community in March could not be estimated due to insufficient sample size.

Proportion of indoor contact hours by location

We estimated the proportion of contact hours occurring within the homes (Context 1) and outside the communities (Context 3). Contact hours in transport were excluded in the Context 3 proportions due to the uncertainty surrounding the proportion of contacts with individuals from within or outside the community. However, the small proportion of contact time that occurred in transport (Fig 4 in the main results), is unlikely to have substantially influenced these results. Furthermore, while participants were asked to provide details on the origin and destination of each transport journey, these data were only available for KZN participants (Table S6 in S1 Appendix).

Table S6 in S1 Appendix. Number and proportion of journeys based on origin and destination among peri-urban and rural participants.

	Destination		
	To inside	To outside	To unknown location
**Origin**	**n (%)**	**n (%)**	**n (%)**
**Peri-urban community**			
From inside	284 (63.4%)	72 (16.1%)	3 (0.67%)
From outside	70 (15.6%)	18 (4.02%)	0 (0%)
From unknown location	0 (0%)	1 (0.223%)	0 (0%)
**Rural community**			
From inside	52 (19.8%)	100 (38.2%)	1 (0.382%)
From outside	80 (30.5%)	25 (9.54%)	1 (0.382%)
From unknown location	2 (0.763%)	0 (0%)	1 (0.382%)

Tables S7-1:3 in S1 Appendix display the proportion of contact hours per day occurring in buildings inside and outside households and communities. These results correspond to Fig 3 in the main results.

Table S7-1 in S1 Appendix. Proportion of contact hours inside and outside the household and community among urban participants.

**Urban community**								
	**Inside household**		**Outside household**		**Inside community**		**Outside community**	
**Strata**	**Proportion**	**95% CI**	**Proportion**	**95% CI**	**Proportion**	**95% CI**	**Proportion**	**95% CI**
**Total**	45.9%	(42.9-49.0%)	54.1%	(51.0-57.1%)	31.8%	(27.6-36.5%)	68.2%	(63.5-72.4%)
**Sex**								
Female	51.4%	(46.9-55.8%)	48.6%	(44.2-53.1%)	32.3%	(26.0-38.9%)	67.7%	(61.1-74.0%)
Male	39.6%	(35.7-44.0%)	60.4%	(56.0-64.3%)	31.4%	(25.3-37.6%)	68.6%	(62.4-74.7%)
**Age group**								
18-24	45.2%	(40.1-51.2%)	54.8%	(48.8-59.9%)	51.1%	(41.4-60.4%)	48.9%	(39.6-58.6%)
25-44	44.1%	(40.1-47.9%)	55.9%	(52.1-59.9%)	22.8%	(18.5-27.4%)	77.2%	(72.6-81.5%)
45+	60.0%	(50.5-70.5%)	40.0%	(29.5-49.5%)	32.0%	(17.1-50.3%)	68.0%	(49.7-82.9%)
**Employment**								
Full-time	34.2%	(30.2-38.3%)	65.8%	(61.7-69.8%)	16.6%	(11.9-22.1%)	83.4%	(77.9-88.1%)
Part-time	40.7%	(34.9-47.4%)	59.3%	(52.6-65.1%)	15.7%	(10.5-21.7%)	84.3%	(78.3-89.5%)
Unemployed	61.5%	(56.7-66.4%)	38.5%	(33.6-43.3%)	71.1%	(63.1-78.0%)	28.9%	(22.0-36.9%)

Table S7-2 in S1 Appendix. Proportion of contact hours inside and outside the household and community among peri-urban participants.

Peri-urban community								
	Inside household		Outside household		Inside community		Outside community	
Strata	Proportion	95% CI	Proportion	95% CI	Proportion	95% CI	Proportion	95% CI
**Total**	70.8%	(67.6-73.8%)	29.2%	(26.2-32.4%)	77.9%	(71.9-83.9%)	22.1%	(16.1-28.1%)
**Sex**								
Female	74.9%	(70.9-78.9%)	25.1%	(21.1-29.1%)	80.3%	(72.3-87.7%)	19.7%	(12.3-27.7%)
Male	64.6%	(59.6-69.3%)	35.4%	(30.7-40.4%)	75.5%	(67.1-84.1%)	24.5%	(15.9-32.9%)
**Age group**								
18-24	66.1%	(58.2-73.8%)	33.9%	(26.2-41.8%)	87.3%	(78.1-95.5%)	12.7%	(4.5-21.9%)
25-44	71.1%	(66.0-75.4%)	28.9%	(24.6-34.0%)	80.2%	(71.9-88.3%)	19.8%	(11.7-28.1%)
45+	72.4%	(66.9-77.1%)	27.6%	(22.9-33.1%)	70.3%	(58.9-81.0%)	29.7%	(19.0-41.1%)
**Employment**								
Full-time	63.7%	(58.3-69.4%)	36.3%	(30.6-41.7%)	70.8%	(60.0-81.2%)	29.2%	(18.8-40.0%)
Part-time	66.2%	(49.6-81.5%)	33.8%	(18.5-50.4%)	81.1%	(49.3-100.0%)	18.9%	(1.4-51.1%)
Unemployed	74.6%	(70.8-78.0%)	25.4%	(22.0-29.2%)	82.2%	(74.8-88.9%)	17.8%	(11.1-25.2%)

Table S7-3 in S1 Appendix. Proportion of contact hours inside and outside the household and community among rural participants.

Rural community								
	Inside household		Outside household		Inside community		Outside community	
Strata	Proportion	95% CI	Proportion	95% CI	Proportion	95% CI	Proportion	95% CI
**Total**	76.2%	(73.5-79.0%)	23.8%	(21.0-26.5%)	61.2%	(54.8-68.1%)	38.8%	(31.9-45.2%)
**Sex**								
Female	77.2%	(73.8-80.4%)	22.8%	(19.6-26.2%)	57.3%	(48.1-66.5%)	42.7%	(33.5-51.9%)
Male	74.5%	(69.5-78.7%)	25.5%	(21.3-30.5%)	67.0%	(56.7-76.9%)	33.0%	(23.1-43.3%)
**Age group**								
18-24	65.8%	(59.4-72.4%)	34.2%	(27.6-40.6%)	69.6%	(57.4-80.7%)	30.4%	(19.3-42.6%)
25-44	76.0%	(71.4-80.5%)	24.0%	(19.5-28.6%)	55.2%	(44.0-66.5%)	44.8%	(33.5-56.0%)
45+	81.7%	(77.7-85.4%)	18.3%	(14.6-22.3%)	59.7%	(46.9-72.5%)	40.3%	(27.5-53.1%)
**Employment**								
Full-time	67.0%	(58.1-76.0%)	33.0%	(24.0-41.9%)	60.1%	(40.9-78.2%)	39.9%	(21.8-59.1%)
Part-time	59.0%	(43.2-71.6%)	41.0%	(28.4-56.8%)	38.7%	(19.8-64.7%)	61.3%	(35.3-80.2%)
Unemployed	78.4%	(75.6-81.2%)	21.6%	(18.8-24.4%)	63.4%	(56.0-70.9%)	36.6%	(29.1-44.0%)

Proportion of indoor contact hours by congregate setting

Table S8 in S1 Appendix displays the proportion of indoor contact hours per day occurring outside the household attributable to each congregate setting. These results, including the display order, correspond to Fig 4 in the main results.

Table S8 in S1 Appendix. Proportion of contact hours in congregate settings outside own home by community.

	Urban		Peri-urban		Rural	
**Congregate setting**	**Proportion**	**95% CI**	**Proportion**	**95% CI**	**Proportion**	**95% CI**
Transport	11.7%	(10.0-15.0%)	11.2%	(9.0-14.0%)	10.4%	(8.0-13.0%)
Unknown building	4.0%	(2.0-6.0%)	0.9%	(0.0-3.0%)	0.1%	(0.0-0.1%)
Other building	7.4%	(5.0-9.0%)	13.8%	(10.0-17.0%)	10.7%	(7.0-15.0%)
Food and leisure	15.7%	(13.0-20.0%)	6.0%	(3.0-9.0%)	2.5%	(1.0-4.0%)
Retail and office	19.2%	(14.0-22.0%)	4.6%	(2.0-7.0%)	3.0%	(1.0-5.0%)
Workshop	12.2%	(9.0-16.0%)	12.4%	(8.0-16.0%)	12.6%	(8.0-17.0%)
Community services	9.1%	(7.0-11.0%)	19.7%	(16.0-25.0%)	14.1%	(10.0-19.0%)
School	11.5%	(9.0-15.0%)	15.4%	(11.0-21.0%)	21.4%	(16.0-27.0%)
Other homes	9.1%	(8.0-11.0%)	16.1%	(12.0-21.0%)	25.1%	(20.0-31.0%)

Acknowledgments

The KwaZulu-Natal social contact survey was part of the larger *Umoya omuhle* project,[4] and we express our gratitude to the entire team. Project members are listed alphabetically by surname and are reproduced from McCreesh et al.[5]

Table S9 in S1 Appendix. Umoya omuhle project members.

Name	Institution(s)	Role
Siphokazi Adonisi	UCT	Research Assistant
Kathy Baisley	LSHTM; AHRI	Co-investigator
Peter Beckwith	LSHTM; UCT	Research fellow
Fiammetta Bozzani	LSHTM	Co-investigator
Amy Burdzik	UCT	Occupational health
Adrienne Burrough	LSHTM	Project Manager
Nkosingiphile Buthelezi	AHRI	Research Assistant
Xolile Buthelezi	AHRI	Diagnostic Lab Manager
Ruvimbo Chigwanda	UCT	Administration
Christopher Colvin	UCT	Co-investigator
PIP CRAs	AHRI	Clinic research Assistants
Njabulo Dayi	AHRI	Research Data Manager
Arminder Deol	LSHTM	Mathematical modeller
Karina Diaconu	QMU	Co-investigator
Siphephelo Dlamini	AHRI	Nursing Manager
Yutu Dlamini	AHRI	Research Assistant
Raveshni Durgiah	AHRI	Grants office
Anita Edwards	AHRI	Head: Scientific Support
Jennifer Falconer	QMU	Research Assistant
Kitty Flynn	QMU	Administrator
Patrick Gabela	AHRI	Clinical Research Data Coordinator
Dickman Gareta	AHRI	Head: Research Data Management
Awethu Gawulekapa	UCT	Research Assistant
Harriet Gliddon	AHRI; UCL	Research Assistant
Bavashni Govender	UKZN	Administration
Indira Govender	LSHTM; AHRI	Co-investigator
Alison Grant	LSHTM; AHRI	Principal investigator
Meghann Gregg	LSE	Research fellow
Emmerencia Gumede	AHRI	Research Assistant
Sashin Harilall	AHRI	Grants office
Kobus Herbst	AHRI	Chief Information Officer
Tamia Jansen	UCT	Research Assistant
Seonaid Kabiah	UCT	Research Assistant
Idriss Kallon	UCT	Post-doctoral researcher
Aaron Karat	LSHTM	Co-investigator
Hannah Keal	AHRI	Communications
Suzanne Key	UCT	Occupational health
Zama Khanyile	UKZN	Research Assistant
Mandla Khoza	AHRI	Clinic Research Assistant
Nozi Khumalo	AHRI	Systems Engineer
Zilethile Khumalo	AHRI	Research Assistant
Karina Kielmann	QMU	Co-principal investigator
Nondumiso Kumalo	AHRI	Clinic Research Assistant
Richard Lessells	AHRI	Epidemiologist
Nokuthula Lushaba (deceased)	UKZN	Administration
Sithembiso Luthuli	AHRI	Research Assistant
Sinethemba Mabuyakhulu	AHRI	Clinic Research Assistant
Hayley MacGregor	IDS	Co-investigator
Nonhlanhla Madlopha	AHRI	Research Assistant
Aphiwe Makalima	UCT	Administration
Tacha Malaza	AHRI	PIP CRA
Sifundesihle Malembe	AHRI	Research Assistant
Godfrey Manuel	UCT	Transport
Nonhlanhla Maphumulo	UKZN	Administration
Precious Mathenjwa	UCT	Research Assistant

Name	Institution(s)	Role
Sanele Mbuyazi	AHRI	PIP CRA
Nicky McCreesh	LSHTM	Co-investigator
Claire McLellan	QMU	Administrator
Simphiwe Mdluli	AHRI	PIP CRA
Thabile Mkhize	AHRI	Transport
Duduzile Mkhwanazi	AHRI	Research Assistant
Zinhle Mkhwanazi	AHRI	Research Assistant
Zodwa Mkhwanazi	AHRI	Research Assistant
Anathi Mngxekeza	UCT	Research Assistant
Tshwaraganang Modise	AHRI	Research Data
Sashen Moodley	AHRI	Microbiology Laboratory Supervisor
Samantha Moyo	UCT	Research Assistant
Silindile Mthembu	AHRI	Clinic Research Assistant
Nozipho Mthethwa	AHRI	Research Assistant
Siphesihle Mthethwa	AHRI	Procurement Coordinator
Sphiwe Mthethwa	AHRI	Research Assistant
Sanele Mthiyane	AHRI	Research Assistant
Vanisha Munsamy	AHRI	Grants office
Sinead Murphy	UCT	Research Assistant
Thomas Murray	AHRI	Research assistant
Senzile Myeni	AHRI	PIP CRA
Tevania Naidoo	AHRI	Procurement
Nompilo Ndlela	AHRI	Research Assistant
Zama Ndlela	AHRI	PIP CRA
Thandekile Nene	AHRI	Research Assistant
Phumla Ngcobo	AHRI	Communications
Nzuzo Ntombela	AHRI	Research Data Systems Service Manager
Sabelo Ntuli	AHRI	GIS Coordinator
Nompumulelo Nyawo	AHRI	Human resources
Phumzile Nywagi	UCT	Research Assistant
Stephen Olivier	AHRI	Statistician
Justin Parkhurst	LSE	Co-investigator
Alex Pym	AHRI	Co-investigator
Yolanda Qeja	UCT	Research Assistant
Anand Ramnanan (deceased)	AHRI	Procurement
Sharmila Rugbeer	UKZN	Administration
Janet Seeley	LSHTM	Co-investigator
Aruna Sevakram	AHRI	Scientific support
Sizwe Sikhakane	AHRI	Transport
Zizile Sikhosana	AHRI	Somkhele Laboratory Supervisor
Theresa Smit	AHRI	Head: Diagnostic Research
Thandeka Smith	UKZN	Research Assistant
Naomi Stewart	LSHTM	Communications
Alison Swartz	UCT	Co-investigator
Amy Thomas	LSHTM	Communications
Siphosethu Titise	UCT	Research Assistant
Anna Vassall	LSHTM	Co-investigator
Marlise Venter	AHRI	Facilities Administrator
Anna Voce	UKZN	Co-investigator
Richard White	LSHTM	Co-investigator
Tom Yates	Imperial	Co-investigator
Precious Zulu	AHRI	Administration
Gimenne Zwama	QMU	Research Fellow

AHRI: Africa Health Research Institute; IDS: Institute of Development Studies; LSE: London School of Economics and Political Science; LSHTM: London School of Hygiene & Tropical Medicine; QMU: Queen Margaret University; UCT: University of Cape Town; UKZN: University of KwaZulu-Natal

References

1. McCreesh N, Middelkoop K, Mohlamonyane M. Cape Town social contact data [Internet]. London, United Kingdom: Data Compass; 2022. Available from: https://datacompass.lshtm.ac.uk/id/eprint/2756/

2. McCreesh N, Dlamini V, Edwards A, Olivier S, Dayi N, Dikgale K, et al. Social contact information from PIPSA residents in uMkhanyakude before and during the Covid-19 pandemic – data from the Umoya Omuhle and Covid Social Contacts studies [Internet]. AHRI Data Repository; 2021. Available from: https://data.ahri.org/index.php/catalog/1018

3. McCreesh N, Dlamini V, Edwards A, Olivier S, Dayi N, Dikgale K, et al. Impact of the Covid-19 epidemic and related social distancing regulations on social contact and SARS-CoV-2 transmission potential in rural South Africa: analysis of repeated cross-sectional surveys. BMC Infectious Diseases. 2021 Sep 8;21(1):928.

4. London School of Hygiene & Tropical Medicine. Umoya omuhle [Internet]. 2020 [cited 2024 Nov 30]. Available from: https://www.lshtm.ac.uk/research/centres-projects-groups/uo

5. McCreesh N, Karat AS, Govender I, Baisley K, Diaconu K, Yates TA, et al. Estimating the contribution of transmission in primary healthcare clinics to community-wide TB disease incidence, and the impact of infection prevention and control interventions, in KwaZulu-Natal, South Africa. BMJ Global Health. 2022 Apr 1;7(4):e007136.
